# Evaluation of a rapid and automated heparin‐induced thrombocytopenia immunoassay

**DOI:** 10.1111/ijlh.13029

**Published:** 2019-04-15

**Authors:** Majed A. Refaai, Grace Conley, Thomas L. Ortel, John L. Francis

**Affiliations:** ^1^ Department of Pathology and Laboratory Medicine University of Rochester Medical Center Rochester New York; ^2^ Hemostasis and Thrombosis Center Duke University Medical Center Durham North Carolina; ^3^ Florida Hospital Center for Thrombosis Research Winter Park Florida

**Keywords:** heparin, heparin‐induced thrombocytopenia, platelets, thrombocytopenia, thrombosis

## Abstract

**Introduction:**

Heparin‐induced thrombocytopenia (HIT) is a potentially life‐threatening adverse reaction of heparin. Laboratory evaluation of HIT is often not available within a reasonable time. We evaluated the HemosIL^®^ HIT‐Ab_(PF4‐H)_ (Instrumentation Laboratory), a rapid, on‐demand, fully automated, latex immunoturbidimetric assay (LIA).

**Materials and methods:**

Following determination of the LIA's reference interval and cutoff values, a multicenter study was conducted between March 2013 and June 2015. Plasma samples of HIT‐suspected patients (n = 632) were collected and evaluated by LIA on the ACL TOP^®^ Family systems (Instrumentation Laboratory), enzyme‐linked immunosorbent assays (EIA), and serotonin release assay (SRA). Patient characteristics, medical conditions, comorbidities, laboratory results, and medications were collected via medical chart review. The pretest clinical probability of HIT was also calculated for each patient.

**Results:**

Based on the 95% reference interval for healthy donors and HIT‐negative patients, a LIA value ≥1.0 U/mL was interpreted positive. The overall agreement of LIA versus EIA and SRA results were 90% (95% CI 88%‐92%) and 79% (95% CI 75%‐82%), respectively. The negative predictive value for LIA and EIA was comparable (87%) with SRA. The positive and negative percent agreements with the clinical probability were 89% (95% CI 69%‐97%) and 86% (95% CI 83%‐89%), respectively, with a negative predictive value of 99.6% (95% CI 98%‐100%).

**Discussion:**

Overall, the LIA results were comparable to those of EIA and SRA. This fully automated assay with a remarkable short analytical turnaround time of <20 minutes can be performed on‐demand, which would greatly facilitate more prompt management of HIT.

## 1 INTRODUCTION

Heparin is commonly used in the management of thrombosis and as a thromboprophylaxis measure in many clinical conditions, such as cardiovascular surgery, orthopedic surgery, and during invasive procedures.^1^ Almost one third of the inpatient population in the United States receives some type of heparin during hospitalization (ie, about 12 million patients per year).^2^ One of the main possible complications of heparin therapy is heparin‐induced thrombocytopenia (HIT), which is a potentially life‐threatening, immune complex–mediated adverse reaction. Due to the widespread use of heparin, HIT is considered one of the most common adverse drug reactions, and the most frequent form of immune‐mediated drug‐induced thrombocytopenia. If unrecognized, HIT may be associated with significant morbidity and mortality. Unfortunately, since thrombocytopenia is very common in various medical conditions, a diagnosis of HIT may be missed.

Clinically, HIT may be suspected in patients who develop, otherwise unexplained, significant drop in platelet count (≥50%) and/or development of thrombosis within 5‐10 days after the start of unfractionated heparin (UFH) or low molecular weight heparin (LMWH). Although about 8% of patients receiving heparin are at risk for developing HIT antibodies, only 1%‐5% will develop thrombocytopenia, and approximately one third of these may develop arterial and/or venous thrombosis.^3^, ^4^ The risk of HIT depends on the type of heparin, length of exposure, and patient risk factors. For example, the incidence is higher among patients receiving UFH (almost 10‐fold) versus LMWH^6^ and in patients undergoing major versus minor surgeries.^7^

Heparin forms complexes with platelet factor‐4 (PF4), a circulating plasma protein that is mainly secreted by platelets, in vivo. The immunogenicity of these glycoprotein structures results in the rapid generation of IgG antibodies (between day 5 and 14),^8^ which, in turn, bind to the PF4‐heparin complex, forming PF4‐heparin‐IgG immune complexes. These immune complexes crosslink FcγRIIa receptors on the membranes of platelets^9^ and monocytes (FcγRI)^10^, ^11^ causing their activation that, along with possible alterations of endothelial cells,^12^ promotes thrombin generation.

Typically, the platelet count in HIT starts to fall 5‐10 days^13^ after the initiation of heparin therapy; however, in case of a previous exposure (within 90 days), thrombocytopenia may evolve within minutes to hours of re‐exposure to heparin due to preformed antibodies, resulting in a more rapid‐onset form of HIT.^13^, ^14^ In either case, heparin‐PF4 antibodies may persist in the plasma for an additional 2‐3 months.^4^

The diagnosis of HIT is primarily based on clinical judgment, but since HIT is considered a clinical‐pathological syndrome, this diagnosis should be confirmed by appropriate laboratory evaluation. Laboratory confirmation of HIT can be achieved by platelet activation (functional) assays, or using immunoassays that can directly detect heparin‐PF4 antibodies. Immunoassays are more commonly performed and are technically less demanding. These assays generally have high sensitivity (80%‐100%) but suffer from low specificity due to the detection of antibodies that are not platelet‐activating, and therefore do not elicit HIT (false positives).^15^ Enzyme‐linked immunosorbent assays (EIA) are most widely used to support the diagnosis of HIT; however, this test platform typically requires sample batching, which may delay confirmation of the diagnosis of HIT, and the EIA may be unavailable in laboratories with low test volumes.

Platelet activation assays for HIT (ie, the serotonin release assay [SRA] and heparin‐induced platelet activation [HIPA]) test measure platelet activity in the presence of the patient's serum and multiple concentrations of heparin. These assays are more specific than EIAs for clinically relevant HIT antibodies. However, these assays are technically demanding and time‐consuming. They are therefore restricted to specialized laboratories and are usually utilized after positive immunoassay results have been obtained, further delaying confirmation or exclusion of the diagnosis.

Since laboratory test results are often not available within a reasonable time (usually hours to days), it is essential to discontinue all heparin exposure in strongly suspected HIT patients, including heparin‐coated catheters and heparin flushes, and initiate alternative anticoagulation. If bridging to warfarin has already been started in acute HIT patient, warfarin therapy should be postponed pending thrombocytopenia recovery (platelet count >150 × 10^9^/L)^9^ as warfarin predisposes to microvascular thrombosis.^16^, ^17^ We evaluated the HemosIL^®^ HIT‐Ab_(PF4‐H)_ (Instrumentation Laboratory, Bedford, MA), a rapid, on‐demand, fully automated, latex immunoturbidimetric assay, as an aid in the diagnosis of HIT and provides results more quickly than existing tests. The assay detects HIT antibodies through a reaction mechanism that competitively inhibits agglutination of HIT‐like monoclonal antibody‐bearing particles.^18^

## 2 MATERIALS AND METHODS

### 2.1 Study design

This is a cohort study to investigate the diagnostic accuracy of a newly developed automated, on‐demand HIT assay HemosIL^®^ HIT‐Ab_(PF4‐H)_ (Instrumentation Laboratory). In addition, we aimed to demonstrate the substantial equivalence of this latex immunoturbidimetric assay (LIA) to a reference method (Serotonin Release Assay) and to a predicate method (Stago Asserachrom HPIA‐IgGAM ELISA assay, Diagnostica Stago, Asnieres, France).

### 2.2 Patient population

A multicenter study was conducted between March 2013 and June 2015. Hospitalized patients with a clinical suspicion of a HIT diagnosis, as defined by the institution's guidelines, were included in this evaluation. The protocol was approved by the Ethics Committees of the three participating medical centers (University of Rochester Medical Center, Rochester, NY; Duke University Medical Center, Durham, NC; and Florida Hospital, Orlando, FL). The study received exemption from informed consenting as previously frozen leftover plasma samples were exclusively used in this evaluation. Patients treated with unfractionated heparin (UFH) or low molecular weight heparin (LMWH) were included. The 4T pretest probability scores were either obtained from the relevant patient record, or were calculated before or after testing by the Principal Investigator at each study site. Patients previously diagnosed with HIT, but not currently under suspicion of recurrence, were excluded. Patients with missing information needed to calculate the 4T pretest probability score were also excluded.

Patient's medical record information including demographic characteristics (sex, age, race, and ethnicity), medical conditions, comorbidities, medications, provenance (cardiovascular surgery, orthopedic surgery, intensive care units, etc), type of heparin (UFH, LMWH), and the original clinical HIT antibody test result was collected via medical chart review.

**Figure 1:**
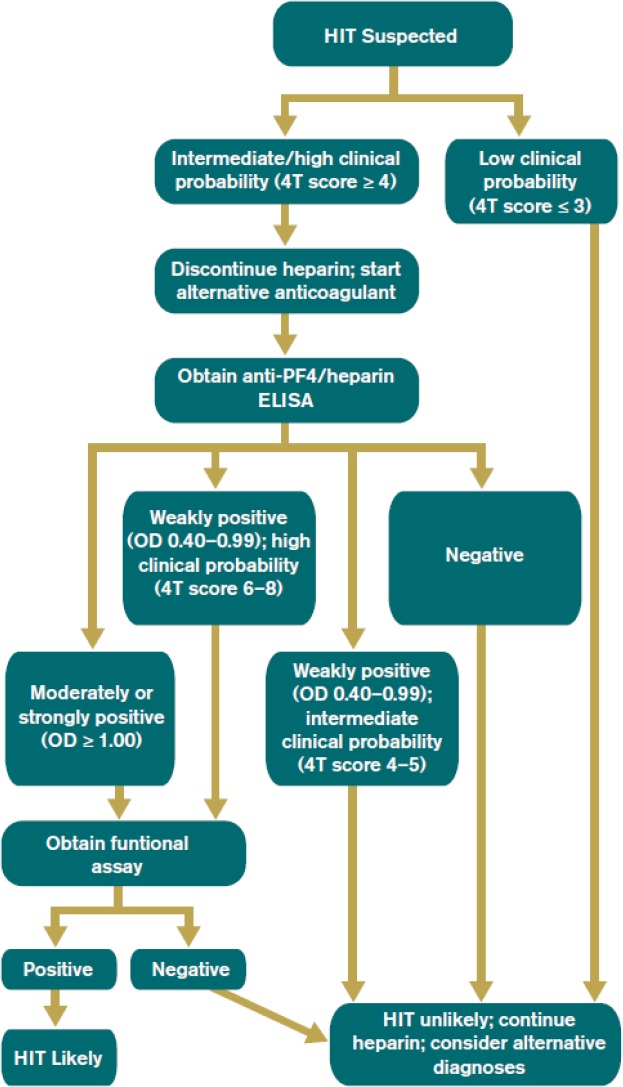
The 2013 American Society of Hematology (ASH) guideline (29). To define the clinical probability of HIT (HIT likely or HIT unlikely) using the 4Ts score, EIA results, and SRA results [Colour figure can be viewed at http://www.wileyonlinelibrary.com]

The pretest clinical probability of HIT was calculated for each patient in the study using the 4T Score HIT Assessment Point System.^19^ Additionally, the overall clinical probability was calculated using the 2013 American Society of Hematology (ASH) guideline that was adopted in part from the 9th edition of the American College of Chest Physicians Evidence‐Based Clinical Practice Guidelines,^20^ a diagnostic algorithm that classifies samples as “HIT Likely” or “HIT Unlikely” based on the “4Ts” score, EIA, and SRA results (Figure 1).

### 2.3 Sample handling and processing

As part of routine clinical care, a freshly collected (within 4 hours) sodium citrated sample was obtained from each HIT‐suspected patients at time of HIT testing at each center. Leftover plasma samples that were no longer needed for clinical testing were processed, and three aliquots (500 L each) were generated for each sample and kept frozen at a minimum of −70°C until testing. Samples were then rapidly thawed at 37°C, or per institution's process for thawing, centrifuged at 1500 xg to remove precipitates and homogenized adequately prior to testing. Hemolyzed, icteric, lipemic, partially clotted, and/or turbid samples were excluded as interferences of this assay were determined by the manufacturer at the following levels: free hemoglobin >500 mg/dL, bilirubin >19 mg/dL, and triglycerides >375 mg/dL. Samples were de‐identified, and a unique study ID was assigned to each enrolled study specimen prior to study‐related testing. All samples were tested following only a single freeze‐thaw cycle when possible.

Samples were tested by trained clinical laboratory technologists using the LIA on the ACL TOP Family system (Instrumentation Laboratory) and by EIA (Asserachrom^®^ HPIA assay; predicate assay; n = 632; see below). Tests were performed in duplicate according to the manufacturer's package insert for the predicate assay. An aliquot of each sample was also shipped on dry ice to the Florida Hospital Center for Thrombosis Research (Winter Park, FL) for SRA testing (n = 537).

### 2.4 Serotonin release assay

The SRA was performed as previously described^21^ to detect heparin‐dependent platelet‐activating anti‐PF4/heparin antibodies. Briefly, following the collection of citrated whole blood, platelet‐rich plasma was prepared via differential centrifugation. Platelets were then washed in calcium‐ and albumin‐free Tyrode's buffer and radioactively labeled with 14C‐serotonin and incubated with heat‐inactivated patient plasma or control plasma. Samples were tested in duplicate with 5 L low (final concentration of 0.1 and 0.3 IU/mL) and high (100 IU/mL) concentrations of unfractionated heparin (UFH). The release of 14C‐serotonin was measured in a scintillation counter (Packard, Top‐count, Meriden, CT, USA). A release of ≥20% with lower concentrations of UFH and an inhibition of >50% with the higher concentrations of UFH was considered positive. All SRA assays were performed at a single center Florida Hospital Center for Thrombosis Research, Winter Park, FL.

### 2.5 HemosIL^®^ HIT‐Ab_(PF4‐H)_ assay

As previously described,^18^ this latex immunoturbidimetric assay (LIA) uses polystyrene latex nanoparticles coated with a HIT‐mimicking anti‐PF4/heparin murine monoclonal antibody.^22^ Mixing these particles with complexes of PF4/polyvinylsulfonate (PVS) causes agglutination that increases the absorbance. This agglutination is inhibited by the addition of patient plasma containing PF4/heparin‐reactive antibodies, resulting in little to no absorbance increase. The antibody concentration (expressed in units per milliliter) in the sample is therefore inversely proportional to the increase in absorbance. The assay is calibrated using dilutions of the same antibody used to coat the latex particles. The results are expressed as U/mL. All LIA tests were performed in singlicate according to the manufacturer's instructions on the ACL TOP^®^ Family systems (Instrumentation Laboratory). Of note, LIA assay can only be performed on plasma samples.

### 2.6 Cutoff determination

#### 2.6.1 Determining the assay reference interval

##### Reference interval in healthy donors

Venous blood samples were collected from 131 apparently healthy volunteers into a 4.5‐mL blue‐top tube containing 3.2% buffered sodium citrate. Tubes were gently inverted immediately following collection to promote mixing. Plasma samples were then processed and analyzed by LIA on ACL TOP^®^ Family systems (Instrumentation Laboratory) and EIA (Asserachrom HPIA assay, Diagnostica Stago).

##### Reference interval in heparin‐exposed and hit‐suspected patients (HIT negative)

For this group, samples were collected from patients who were exposed to heparin and suspected of having HIT but had tested negative by either of the commercially available EIAs (Asserachrom HPIA assay or and GTI‐PF4 Enhanced EIA, Immucor GTI Diagnostics, Inc, Waukesha, WI). As above, citrated whole blood samples (4.5 mL) were collected from 122 patients. Samples were then processed and analyzed by LIA utilizing ACL TOP Family systems.

### 2.7 Receiver operating characteristic curve analysis

A method comparison study with the SRA was used to confirm the LIA cutoff value by Receiver operating characteristic (ROC). Sixty‐three frozen plasma samples from HIT‐suspected patients with low, moderate, and high 4Ts score (31 were confirmed positive and 32 were confirmed negative by SRA) were analyzed by LIA utilizing ACL TOP Family systems.

### 2.8 Statistical analysis

Reference interval studies data were analyzed using Microsoft Excel 2010 (version 14.0) and the data analysis add‐on package Analyse‐It (version 3.90.1). The reference interval for each study was then determined by nonparametric quantiles. The normal donors' reference interval was performed using the 3rd edition of the Clinical and Laboratory Standards Institute (CLSI) EP28‐A3 guidelines. Partitioned reference intervals were shown to be unnecessary using the Student *t* method with two‐sided 95% confidence interval (CI) to compare the central locations of the distribution of LIA results across gender. The reference interval of the heparin‐exposed and HIT‐suspected patients (HIT Negative) was calculated according to the CLSI EP28‐A3c guidelines. ROC curve analysis was performed using CLSI EP24‐A2 bias estimation guidelines. The data were also analyzed using Microsoft Excel and the data analysis add‐on package Analyse‐It. The ninety‐five percent (95%) CI for total agreement of LIA vs SRA was calculated using Wilson's score confidence interval.

The clinical studies data were analyzed using Microsoft Excel 2010 and the data analysis add‐on package Analyse‐It. Pearson chi‐squared tests were performed to evaluate differences in gender distribution across sites (*P* = 0.9596) and to ensure equality of the test outcome (Positive/Negative) distribution across sites (LIA: *P* = 0.2320, EIA: *P* = 0.8880, SRA: *P* = 0.3149, clinical probability: *P* = 0.7293). The analysis showed that the data from each center could be pooled, and tables are therefore presented for the pooled population. Ninety‐five percent (95%) confidence intervals for positive percent agreement (PPA), negative percent agreement (NPA), and total agreement were calculated using Wilson's score confidence interval. Ninety‐five percent (95%) CI for negative predictive value (NPV) and positive predictive value (PPV) were calculated using Mercado‐Wald's logit confidence interval.

## 3 RESULTS

### 3.1 Determination of the HemosIL HIT‐Ab_(PF4‐H)_ assay reference interval

**Figure 2:**
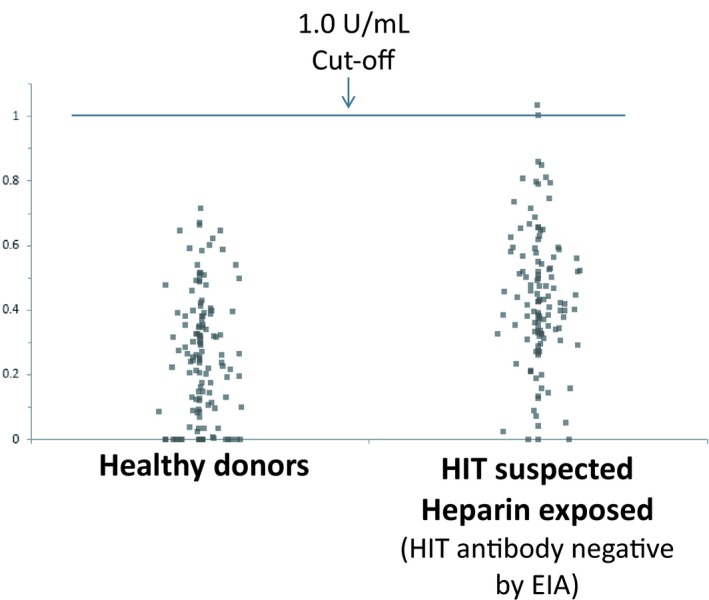
The HemosIL HIT‐Ab_(PF4‐H) _assay Reference Interval. The cutoff was determined by testing blood samples of healthy donors (n‐131) that were tested negative for HIT by EIA; and HIT‐suspected patients (n = 122) that were exposed to heparin but were confirmed negative for HIT antibody by both of the commercially available EIAs (Asserachrom HPIA assay and GTI‐PF4 Enhanced EIA, Immucor GTI Diagnostics, Inc, Waukesha, WI). The 95% reference interval was 0‐0.7 for the healthy donors and 0‐0.9 for the HIT‐suspected patients

The reference interval was calculated using citrated plasma samples from normal donors (n = 131) and HIT‐suspected patients (n = 122) that were confirmed negative by the clinical laboratory by either EIAs. The 95% reference interval for healthy donors was determined to be 0.0‐0.7 U/mL. In HIT‐suspected patients, the 95% reference interval was found to be 0.0‐0.9 U/mL. Thus, based on these results, a LIA value of equal or greater than 1.0 U/mL was interpreted as a positive result for the presence of HIT antibodies (Figure 2).

### 3.2 Receiver operating characteristic curve analysis

**Figure 3:**
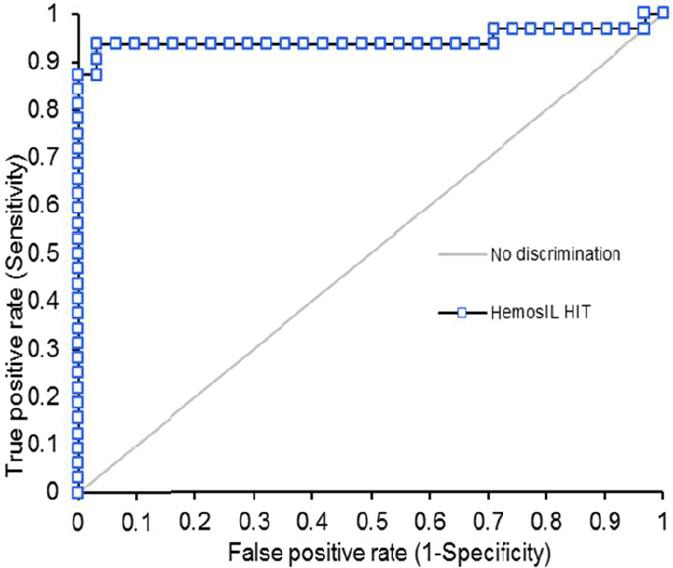
The receiver operating characteristic (ROC) curve analysis of the HemosIL HIT‐Ab_(PF4‐H) _assay versus SRA using 63 frozen plasma samples from HIT‐suspected patients with moderate to high 4Ts score (31 were confirmed positive with SRA and 32 were confirmed negative by SRA) [Colour figure can be viewed at http://www.wileyonlinelibrary.com]

The optimal cutoff value confirmed by ROC analysis was 1.0 U/mL. The ROC curve showed the pattern of an informative assay, and the area under the curve (AUC) was calculated to be 0.95 (95% CI 0.87‐1.0). Total agreement, NPV, and PPV at this cutoff value were 95.2% agreement (95% CI 86.7‐99.0), 93.8% NPV (95% CI 79.2‐99.2), and 96.8% PPV (95% CI 83.3‐99.9), respectively (Figure 3).

### 3.3 Clinical evaluation

The overall agreement of LIA versus EIA and SRA results were 90% (95% CI 88%‐92%), and 79% (95% CI 75%‐82%), respectively (Tables 1 and 2). The negative predictive value for LIA and EIA was comparable (87%) with respect to SRA (Tables 2 and 3). The positive and negative percent agreements of the clinical probability compared to LIA results were 89% (95% CI 69%‐97%) and 86% (95% CI 83%‐89%), respectively, with a NPV of 99.6% (95% CI 98%‐100%; Table 4).

**Table 1:** HemosIL HIT‐Ab_(PF4‐H) _Assay vs EIA (n = 632)

		EIA results		
		+	−	Total
HemosIL HIT‐Ab_(PF4‐H)_ Results	+	67	39	106
	−	22	504	526
	Total	89	543	632

HemosIL HIT_(PF4‐H)_ vs EIA	Proportion	Wilson 95% CI	
PPA (positive percent agreement)	75% (67/89)	65%	83%
NPA (negative percent agreement)	93% (504/543)	90%	95%
Total percent agreement	90% (571/632)	88%	92%

**Table 2:** HemosIL HIT‐Ab_(PF4‐H) _Assay versus SRA. A subset of the study population (n = 537) was compared against SRA

		SRA results		
		+	−	Total
HemosIL HIT‐Ab_(PF4‐H)_ results	+	33	56	89
	−	59	389	448
	Total	92	445	537

HemosIL HIT_(PF4‐H)_ vs SRA	Proportion	Wilson 95% CI	
PPV (positive predictive value)	37% (33/89)	28%	47%
NPV (negative predictive value)	87% (389/448)	83%	90%
Total percent agreement	79% (422/537)	75%	82%

**Table 3:** EIA vs SRA (n = 537)

		SRA results		
		+	−	Total
EIA results	+	31	37	68
	−	61	408	469
	Total	92	445	537

EIA vs SRA	Prop̘ortion	Wilson 95% CI	
PPV (positive predictive value)	46% (31/68)	34%	57%
NPV (negative predictive value)	87% (408/469)	84%	90%

**Table 4:** HemosIL HIT‐Ab_(PF4‐H) _vs clinical probability (20)

		Clinical probability		
		HIT likely	HIT unlikely	Total
HemosIL HIT‐Ab_(PF4‐H)_ results	+	17	72	89
	−	2	446	448
	Total	19	518	537

Clinical Probability according to ASH 2013	Proportion	Wilson 95% CI	
PPA (positive percent agreement)	89% (17/19)	69%	97%
NPA (negative percent agreement)	86% (446/518)	83%	89%
PPV (positive predictive value)	19% (17/89)	12%	28%
NPV (negative predictive value)	99.6% (446/448)	98%	100%

Comparing LIA to SRA after sorting the patients into three different groups (Supplementary Table S1) according to their pretest clinical probability of HIT (ie, “4T” score, low: 1‐3; moderate: 4‐5; or high: 6‐8) revealed better PPA and PPV in the high 4Ts score group (78.6% and 55%, respectively). These results also corresponded to the Asserachrom HPIA EIA assay when compared to the SRA (85.7% and 57.1%, respectively; supplementary Table S2).

## 4 DISCUSSION

This evaluation showed that the LIA is comparable to the Asserachrom HPIA EIA with a total percent agreement of 90%. When compared to the SRA, both methods showed high total percent agreement (79% and 82%, respectively) and an identical NPV of 87%. Utilizing the clinical probability of HIT as a diagnostic tool for HIT, the NPV of the LIA approached 100%, which demonstrates the clinical utility of this assay in excluding a diagnosis of HIT with the appropriate clinical pretest probability.

When patients were sorted into three different groups according to their pretest clinical probability of HIT, the observed PPA and PPV suggest that the LIA exhibits high specificity with an acceptable sensitivity, especially in patients with high “4T” scores. These findings indicate that patients with a high clinical suspicion of HIT may benefit from the earlier HIT diagnosis and management facilitated by this assay. Based upon the current and prior evaluations of the LIA, this rapid assay may also be appropriate for patients with moderate and low pretest probability of HIT.^18^, ^23^

Timing is critical in the diagnosis of HIT and may affect patient management and clinical decisions in HIT‐suspected patients. Even in laboratories that perform the EIA daily, the use of batch testing can still result in turnaround times up to 30 hours.^24^ The advantage of an on‐demand, automated assay is that test results can be provided in a more timely fashion, even in a routine clinical laboratory setting.

The “4T”score was introduced in 2003^25^ and is a commonly used means of predicting the pretest likelihood of clinical HIT using a scale of 0‐8 points. If the pretest probability score falls between 0 and 3, HIT is clinically unlikely; a score of 4‐5 indicates intermediate probability, while a score of 6‐8 makes HIT diagnosis significantly more likely. Patients with moderate and high 4T scores may need to be treated with alternative anticoagulation while appropriate laboratory tests for HIT antibodies are performed to confirm or exclude the clinical diagnosis. Patients with a low score can safely continue receiving heparin as the likelihood of HIT is extremely low^26^, ^27^; however, continued platelet count monitoring is required as the risk of HIT may increase with longer use of heparin.^30^, ^31^

Refaai et al^30^ reported a significant correlation between the HIT antibody EIA optical density (OD) values below the cutoff (ie, negative) and the possibility of clinical development of HIT a few days later when heparin therapy was continued.^30^ Using serial EIA tests, these authors showed that 43% of patients had positive results in the repeat test if the negative titer was ≥66.7% of the assay cutoff (*P* = 0.0026).^30^ Thus, reporting the OD of the HIT EIA, rather than just a negative or positive result according to the cutoff, might better help clinicians predict which patients have a high risk of developing HIT despite a negative initial test. Knowledge of the EIA OD also increases the specificity of a positive result without compromising sensitivity,^32^, ^33^ a finding which has since been confirmed for the LIA.^18^ Therefore, reporting the quantitative values of LIA along with the positive/negative results may help clinicians better assess the posttest probability of HIT and provide the appropriate management at an earlier time.

### 4.1 Conclusions

In summary, the LIA test for HIT antibodies showed comparable results to the commercially available EIA and SRA. This rapid, fully automated laboratory assay for detection of HIT antibodies can be performed on‐demand in any routine coagulation laboratory equipped with an ACL TOP Family analyzer. The remarkably short analytical turnaround time of less than 20 minutes (instrument testing time is 13 minutes) would greatly facilitate more prompt and appropriate management of HIT. Implementing LIA could potentially improve the clinical outcome of HIT patients and reduce costs by averting complications and avoiding unnecessary, more expensive anticoagulant therapy. Further cost effectiveness evaluations are warranted**.**

## CONFLICT OF INTEREST

The authors have no competing interests.
