# Supplementary material for: Evaluation of a rapid and automated heparin‐induced thrombocytopenia immunoassay
Source: Int J Lab Hematol. 2019 Apr 15;41(4):478–84. doi: 10.1111/ijlh.13029 (PMC6850659; doi:10.1111/ijlh.13029)
Supplement: Supplementary file 1 [file IJLH-41-478-s001.docx]

**Supplementary Table 1A:**

**Supplementary Table 1:**


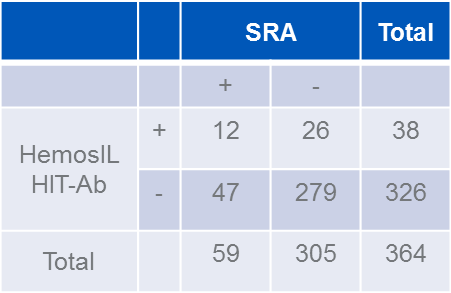

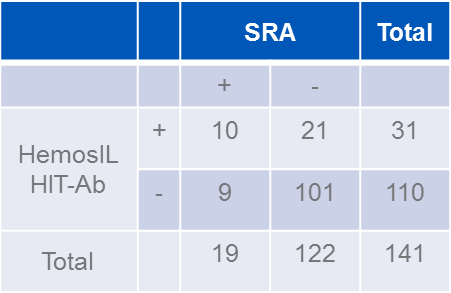

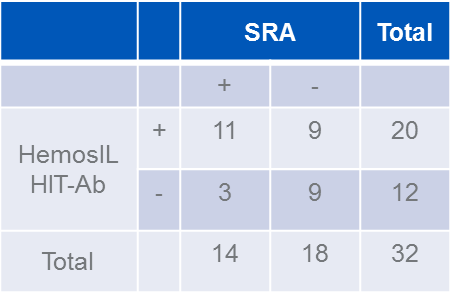


PPA: 20.3% (12.0 – 32.3%)

NPA: 91.5% (87.8 – 94.1%)

PPV: 31.6% (19.8 – 46.3%)

NPV: 85.6% (83.9 – 87.2%)

Agreement: 79.9%

Low 4Ts score

PPA: 52.6% (31.7 – 72.7%)

NPA: 82.8% (75.1 – 88.5%)

PPV: 32.3% (21.1 – 45.9%)

NPV: 91.8% (87.4 – 94.8%)

Agreement: 78.7%

Moderate 4Ts score

PPA: 78.6% (52.4 – 92.4%)

NPA: 50.0% (29.0 – 71.0%)

PPV: 55.0% (41.7 – 67.6%)

NPV: 75.0% (49.9 – 90.1%)

Agreement: 62.5%

High 4Ts score

**Supplementary Table 1B:**

**Supplementary Table 1B:**

**
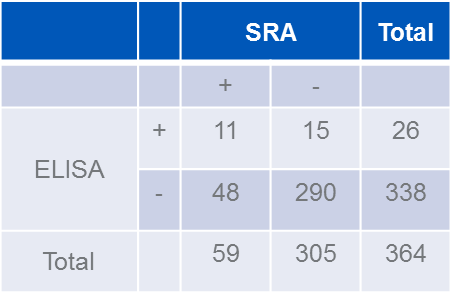

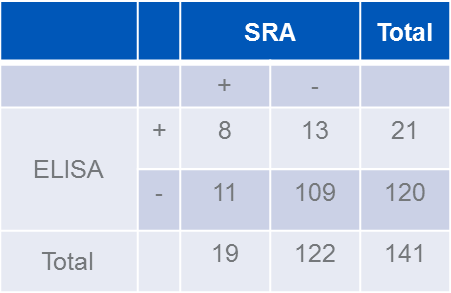

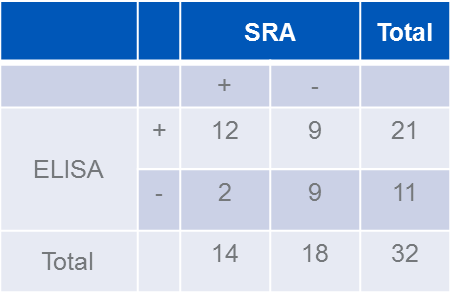
**

PPA: 18.6% (10.7 – 30.4%)

NPA: 95.1% (92.0 – 97.0%)

PPV: 42.3% (26.2 – 60.3%)

NPV: 85.8% (84.2 – 87.3%)

Agreement: 82.7%

Low 4Ts score

PPA: 42.1% (23.1 – 63.7%)

NPA: 89.3% (82.6 – 93.7%)

PPV: 38.1% (22.8 – 56.2%)

NPV: 90.8% (87.0 – 93.6%)

Agreement: 83.0%

Moderate 4Ts score

PPA: 85.7% (60.1 – 96.0%)

NPA: 50.0% (29.0 – 71.0%)

PPV: 57.1% (44.5 – 68.9%)

NPV: 81.8% (53.5 – 94.6%)

Agreement: 65.6%

High 4Ts score
